# Polyphenol-Rich Diets Exacerbate AMPK-Mediated Autophagy, Decreasing Proliferation of Mosquito Midgut Microbiota, and Extending Vector Lifespan

**DOI:** 10.1371/journal.pntd.0005034

**Published:** 2016-10-12

**Authors:** Rodrigo Dutra Nunes, Guilherme Ventura-Martins, Débora Monteiro Moretti, Priscilla Medeiros-Castro, Carlucio Rocha-Santos, Carlos Renato de Oliveira Daumas-Filho, Paula Rego Barros Bittencourt-Cunha, Karina Martins-Cardoso, Cecília Oliveira Cudischevitch, Rubem Figueiredo Sadok Menna-Barreto, José Henrique Maia Oliveira, Desiely Silva Gusmão, Francisco José Alves Lemos, Daniela Sales Alviano, Pedro Lagerblad Oliveira, Carl Lowenberger, David Majerowicz, Ricardo Melo Oliveira, Rafael Dias Mesquita, Georgia Correa Atella, Mário Alberto Cardoso Silva-Neto

**Affiliations:** 1 Laboratório de Sinalização Celular, Programa de Biologia Molecular e Biotecnologia, Instituto de Bioquímica Médica Leopoldo de Meis, Universidade Federal do Rio de Janeiro, Rio de Janeiro, Rio de Janeiro, Brazil; 2 Instituto Nacional de Ciência e Tecnologia em Entomologia Molecular, Rio de Janeiro, Brazil; 3 Laboratório de Bioquímica de Lipídios e Lipoproteínas, Programa de Biologia Molecular e Biotecnologia, Instituto de Bioquímica Médica Leopoldo de Meis, Universidade Federal do Rio de Janeiro, Rio de Janeiro, Rio de Janeiro, Brazil; 4 Laboratório de Biologia Celular, Instituto Oswaldo Cruz, Fundação Oswaldo Cruz, Rio de Janeiro, Rio de Janeiro, Brazil; 5 Laboratório de Bioquímica de Artrópodes Hematófagos, Programa de Biologia Molecular e Biotecnologia, Instituto de Bioquímica Médica Leopoldo de Meis, Universidade Federal do Rio de Janeiro, Rio de Janeiro, Rio de Janeiro, Brazil; 6 Laboratório de Biologia, Instituto Federal de Educação, Ciência e Tecnologia Fluminense, Campos dos Goytacazes, Rio de Janeiro, Brazil; 7 Laboratório de Biotecnologia, Universidade Estadual do Norte Fluminense, Campos dos Goytacazes, Rio de Janeiro, Brazil; 8 Instituto de Microbiologia Prof. Paulo de Góes, Universidade Federal do Rio de Janeiro, Prédio do CCS, Bloco I, Rio de JaneiroRio de Janeiro, Brazil; 9 Department of Biological Sciences, Simon Fraser University, Burnaby, British Columbia, Canada; 10 Departamento de Biotecnologia Farmacêutica, Faculdade de Farmácia, Universidade Federal do Rio de Janeiro, Ilha do Fundão, Rio de Janeiro, Rio de Janeiro, Brazil; 11 Laboratório de Bioinformática, Departamento de Bioquímica, Instituto de Química, Universidade Federal do Rio de Janeiro, Rio de Janeiro, Rio de Janeiro, Brazil; University of Notre Dame, UNITED STATES

## Abstract

**Background:**

Mosquitoes feed on plant-derived fluids such as nectar and sap and are exposed to bioactive molecules found in this dietary source. However, the role of such molecules on mosquito vectorial capacity is unknown. Weather has been recognized as a major determinant of the spread of dengue, and plants under abiotic stress increase their production of polyphenols.

**Results:**

Here, we show that including polyphenols in mosquito meals promoted the activation of AMP-dependent protein kinase (AMPK). AMPK positively regulated midgut autophagy leading to a decrease in bacterial proliferation and an increase in vector lifespan. Suppression of AMPK activity resulted in a 6-fold increase in midgut microbiota. Similarly, inhibition of polyphenol-induced autophagy induced an 8-fold increase in bacterial proliferation. Mosquitoes maintained on the polyphenol diet were readily infected by dengue virus.

**Conclusion:**

The present findings uncover a new direct route by which exacerbation of autophagy through activation of the AMPK pathway leads to a more efficient control of mosquito midgut microbiota and increases the average mosquito lifespan. Our results suggest for the first time that the polyphenol content and availability of the surrounding vegetation may increase the population of mosquitoes prone to infection with arboviruses.

## Author Summary

The dramatic climate changes currently occurring on our planet may likely influence the biology and the distribution of mosquitoes. *Aedes aegypti* is a major vector of arboviruses. However, females feed on plants for a few days before feeding on blood for the first time. Plants are sessile and cannot move to search for better environmental conditions. In times of extreme temperatures, drought, or UV radiation plants produce polyphenols that allow plants to survive under such extreme conditions. Polyphenol ingestion by mosquitoes enhances the activity of an enzyme named AMP-activated protein kinase (AMPK). Mosquitoes emerging from pupae leave the aquatic environment and are readily colonized by many bacterial strains. Polyphenol-fed mosquitoes display a huge activation of AMPK which enhances autophagy in the midgut cells that increases the capture and killing of midgut bacteria, thus enhancing vector lifespan. Therefore, early meals on polyphenol sources allow for the regulation of vector microbiota. These data may provide the basis of a novel strategy that promotes bacterial proliferation and a shortening of vector lifespan.

## Introduction

Arboviruses are the leading vector-borne diseases worldwide. Dengue infections alone affect 50–100 million people. The lack of drugs or vaccines offers no hope that this scenario will change in the near future [1, 2]. Reducing vector populations has been the only effective way to control dengue incidence and fatality rates.

Soon after their eclosion from laid eggs, mosquitoes embark on a very complex life cycle that ultimately gives rise to adults. Female mosquitoes often feed on plants, to obtain carbohydrates, before feeding on blood that may contain pathogens [3]. Pathogen infection will proceed and in the next blood meal transmission to the next host will take place. Several strategies aimed at blocking pathogen transmission have failed to block the spread of such pathogens among human populations. Thus an alternative strategy would be to decrease the lifespan of the blood-feeding stage of the mosquitoes. Transmission could be blocked if the very first blood meal could be avoided. In this case molecules incorporated into mosquito meals in the early days of their adult life could eventually affect their blood-feeding behavior. In the case of *Aedes aegypti*, a major vector for Dengue, Chikungunya and Zika, the first days of adult life are metabolically driven to obtain sugar-based diets from plants. The effect of such meals on vector biology remains to be demonstrated.

Vectorial capacity, the ability of mosquitoes to transmit parasites or pathogens, depends on several factors, including vector competence, mosquito population density, flight capacity, host preferences, biting rate, mosquito immunity and lifespan [4–6]. *Aedes aegypti* is the most important global vector of dengue viruses. The first days in the adult life of *A*. *aegypti* are spent ingesting and obtaining energy from nectar, fruit sugars and phloem sap. Therefore, plant community structure and the access to nectar in the vicinity of pupal eclosion can greatly affect vectorial capacity. Some pathogens have been shown to increase nectar-seeking behaviour following infection [7,8]. However, the potential roles of plant-derived molecules in mosquito metabolism, immunity, lifespan, and pathogen transmission have not been evaluated. Plant nectar and sap are sources of polyphenols, compounds that have been shown to cause many metabolic changes and to enhance longevity in a wide range of models [9–11]. Most phenolic compounds are toxic to insects [12]. Polyphenols produced from decomposing leaf-litter act as larvicides to most mosquitoes [13]. A close biochemical and phylogenetical connection was established between polyphenol content and mosquito larvicidal activity of several plants [14–16]. However, most of these studies are conducted with larval i.e. aquatic stages of developing mosquitoes. It was recently demonstrated that plant phenolics are not toxic to insects in general due to the detoxifing activity of prophenoloxidases [17]. The ingestion of polyphenols is also related to a decrease in gut microbiota in several models [18–21]. Usually polyphenols induce a down regulation of gut microbiota which is associated with decreased inflammation and a longer lifespan [18–21].

Plant-derived fluids are in fact very complex solutions containing amino acids, proteins, triglycerides, polyphenols and sugars that may yield important metabolic effects with the potential to enhance mosquito survival and vector capability. The production of polyphenols is greatly enhanced under extreme environmental conditions [22], and it is now clear that climate changes substantially affect vector distribution and arbovirus transmission [23]. In most mosquito colonies, insects are reared in the absence of polyphenols. However, in a few cases, mosquitoes are fed on raisins, but the effects of such a diet on overall metabolism and lifespan are unknown. Here, we show that mosquitoes might benefit from polyphenol-rich diets through activation of a hypothetical connection between ingested meals and the establishment of a strong autophagic response that ultimately controls mosquito midgut microbiota while enhancing vector average lifespan.

## Methods

### Ethics statement

All the animal care and experimental protocols were conducted following the guidelines of the institutional care and use committee (Committee for Evaluation of Animal Use for Research at the Federal University of Rio de Janeiro, CAUAP-UFRJ) and the NIH Guide for the Care and Use of Laboratory Animals (ISBN 0-309-05377-3). The protocols were approved by CAUAP-UFRJ under the registry #IBQM067-05/16. Technicians dedicated to the animal facility at the Instituto de Bioquímica Médica Leopoldo de Meis conducted all the aspects related to rabbit husbandry under strict guidelines to ensure the careful and consistent handling of the animals.

### Mosquitoes and infection with dengue virus

Mosquitoes were obtained from colonies of *A*. *aegypti* (Liverpool strain), maintained in the insectary of the Laboratório de Sinalização Celular (LabSiCel) at Instituto de Bioquímica Médica Leopoldo de Meis, UFRJ, Rio de Janeiro, Brazil at 28 ± 2°C, 12:12 h L:D, and 80 ± 5% RH. Mosquitoes (≈200 per cage) were reared in plastic cages (30 cm diameter x 20 cm high; the same cages were used throughout this work). Eggs were placed in pans (25 cm × 40 cm × 3 cm) with 300 mL of deoxygenated filtered water to hatch. Larvae were pooled in groups of ~50 per pan, and 0.1 g of dog food (Pedigree Champion Filhotes) was provided every other day. Pupae were collected daily and transferred into cages (as described above). Cotton soaked in 10% sucrose or other experimental diet solution was provided daily to adults. Cotton balls were kept wrapped in aluminium foil to avoid photo-inactivation of polyphenol solutions. We also conducted experiments with the Rockefeller strain of *A*. *aegypti* a obtained from Campos dos Goytacazes and maintained at the insectary facility of Universidade Estadual do Norte Fluminense (UENF). Dengue virus serotype 2 (New Guinea C strain, DENV-2) was mixed 1∶1 with rabbit blood and used for infections. Infected mosquitoes (20, control and 20, Rv-fed) were collected in each experiment at 7 days post-infection and their midguts were dissected, and then transferred to microcentrifuge tubes containing 500 μl of TRIzol (Invitrogen, USA). Total RNA was extracted and cDNA synthesis and quantitative PCR (qPCR) reactions using primers designed for NS5 (S1 Table) were carried out as described below.

### Lifespan experiments

Mosquitoes (200) were fed *ad libitum* on solutions containing: i) Ctrl—0.05% ethanol plus 10% sucrose; ii) Rv—100 μM Resveratrol plus 0.05% ethanol and 10% sucrose; iii) Epi—100 μM Epi-gallo-catechin-gallate plus 0.05% ethanol and 10% sucrose; iv) Gen—100 μM Genistein plus 0.05% ethanol and 10% sucrose; v) Que—100 μM Quercetin plus 0.05% ethanol and 10% sucrose or vi) AbMix—10 U/mL penicillin, 10 U/mL streptomycin and 15 U/mL gentamicin plus 0.05% ethanol and 10% sucrose. Dead individuals were counted daily. Visual comparisons of survival curves, estimated using the Kaplan-Meier method of Proc Lifetest (SAS Institute 2002), were followed by overall tests (Wilcoxon-Gehan test) of differences between survival curves among groups.

### Quantification of midgut bacteria

All materials used for the handling and dissection of mosquitoes were autoclaved at 121°C for 30 min. Mosquitoes were sanitized with alcohol and sodium hypochlorite to eliminate external contamination. Stereomicroscopes and pipettes were sanitized with 70% ethanol. Three independent isolation assays were performed as previously described [24] using three pools of ten midguts each. Briefly, for surface sterilization, mosquitoes were rinsed sequentially for 1 min each in the following solutions: sodium hypochlorite (1%), ethanol (70%) and sterile phosphate-buffered saline (PBS). Mosquitoes were dissected under a microscope in a double-cavity glass slide containing sterile PBS. Ten midguts were rinsed in sterile PBS and transferred to a 1.5-mL tube containing 100 μL of PBS. The contents in each tube were mixed thoroughly with a pestle and were serially diluted. An aliquot of 100 μL from each tube was transferred to a Petri dish containing Brain Heart Infusion (BHI) and incubated at 28°C for 24 or 48 h. Bacterial isolates were maintained at -70°C in a 15% glycerol solution.

### Assays for Rv killing effect with classical bacteria

A concentration range of 50–500 μM Rv was used to evaluate the antimicrobial potential of Rv against several classical microbiome bacteria isolated from female *A*. *aegypti* midguts: *Enterobacter* sp., *Enterococcus* sp., *Bacillus subtilis*, *Klebsiella* sp., *Serratia* sp., and *S*. *marcescens*. The method used to determine the Rv killing effect was based on international standard methodology M7–A6 (bacteria) and M45 A (fastidious bacteria) CLSI/NCCLS (Clinical and Laboratory Standards Institute) [25]. The Rv killing effect was assessed in 24-well plates by diluting (1: 100) 10 mM Rv in an ethanol stock solution and using 20 μL in 2 mL of Mueller-Hinton (MH) or BHI culture medium for each bacterial strain. Microplate wells were inoculated with 1 x 10^4^ CFU/mL of *Enterobacter* sp., *Enterococcus* sp., *Klebsiella* sp., *Serratia* sp., and *S*. *marcescens*, either isolated or mixed, and were incubated overnight at 37°C for all bacteria. Pure medium was used as a negative growth control, and positive controls consisted of inoculated growth medium. After incubation, the determination of the Rv killing effect was based on turbidity measurements (optical density at 600 nm). Rv was diluted in ethanol (EtOH) to allow the pipetting of a homogeneous aliquot from a stock solution. To ensure that this solvent did not influence the results, a control was set up with the same concentration of EtOH used in the assays. Means (n = 3) and standard errors were calculated from at least three independent experiments.

### qPCR analysis of AMP and apoptosis gene expression

Total RNA from 10 midguts and 7 fat bodies was separately extracted from female mosquitoes using TRIzol reagent (Invitrogen, USA), following the manufacturer's instructions. Total RNA (1 μg) was treated with DNAse I (Invitrogen), and first-strand cDNA synthesis was performed using the High-Capacity cDNA Reverse Transcription kit (Applied Biosystems, USA). qPCR was performed in a 7500 Real Time PCR System (Applied Biosystems, USA) using SYBR-GREEN PCR master MIX (Applied Biosystems, USA). All real-time PCR reactions were performed in triplicate using 5 μL of cDNA per reaction. The sequences of the primers and probes used in the qPCR reactions for AMPs (Attacin, Cecropin, Defensin,Gambicin) and genes involved in apoptosis and autophagy (16S, AeDRONC, AeIAP1, CASPASE 16, ARGONAUTE 2 and ATG8), are presented in S1 Table. We used the Delta Ct method for quantification of expression but on S3 Fig where we have used delta Ct.

### RNAi silencing of mosquito AMPK

The role of the AMPK gene in the control of *A*. *aegypti* midgut microbiota was assessed using RNA interference-mediated gene silencing, as described previously [26]. In brief, dsRNAs were constructed using in-vitro transcription with the HiScribe T7 *In Vitro* Transcription Kit (New England Biolabs). Approximately 207 ng of dsRNA was injected into the thorax of 60 cold-anesthetised 2- to 3-day-old female mosquitoes using a nano-injector. The gene silencing efficiency, evaluated using qPCR, was determined by comparison with an unrelated dsRNA-injected group (*Escherichia c*oli gene MalE) 3 days after the dsRNA injection. Typically, 10 mosquitoes were analysed per experiment in each group.

### Evaluation of ROS production in mosquito midguts

ROS levels were assessed according to Oliveira et al. [27]. Briefly, midguts were dissected in PBS at room temperature and were cultured in L-15 medium supplemented with 5% fetal bovine serum without antibiotics. The midguts were then incubated with 5 μM dihydroethidine (DHE) (Invitrogen) for 20 min under dim light, transferred to a glass slide and photographed under a Zeiss AxioObseRver-Z1 equipped with a Zeiss-15 filter set (excitation BP 546/12; beam splitter FT 580; emission LP 590). Fluorescence was quantified with ImageJ software.

### Lipid analysis

Mosquitoes (200 individuals) were fed *ad libitum* for six days in the presence or absence of Rv, Compound C, Compound C + Rv, AICAR, polyphenols or an antibiotic mix. Twenty insects were separately homogenized and subjected to lipid extraction using methanol: chloroform: distilled water (2:1:0.8 v/v). The lipid extracts were analyzed by one-dimensional thin-layer chromatography (TLC) on Silica Gel 60 plates (E. Merck, Darmstadt, Germany) for neutral lipids using n-hexane:diethyl ether:acetic acid (60:40:1 v/v) [28,29]. Cholesterol, cholesteryl-oleate, glycerol-tryoleate, diolein, oleoyl-glycerol and oleic acid (Sigma-Aldrich Co, USA) were used as standards. The lipids were visualized using a charring reagent (Cu_2_SO_4_) after heating at 200°C for 20 min [28–30]. TLCs were scanned, and the densitometry of the bands was assessed using the ImageJ program.

### Western blotting

Seven fat bodies and 10 midguts from females were dissected and homogenized. Protein quantification was performed using the Lowry method [31]. Protein (30 μg) from each sample was separated by SDS–PAGE and immunoblotted with specific antibodies that recognize the phospho-AMPKa subunit protein (1:1000). The ECL method was used for developing following the manufacturer's instructions (GE Lifecare). Low molecular-weight markers used were from Spectra Multicolor Broad Range Protein Ladder SM1841. An antibody against actin was used as a sample loading control (1:10,000). The immunoblots were scanned to quantitate the density of the bands, and the densitometry was analyzed with the ImageJ software.

### Statistical analysis

**Fig 1:**
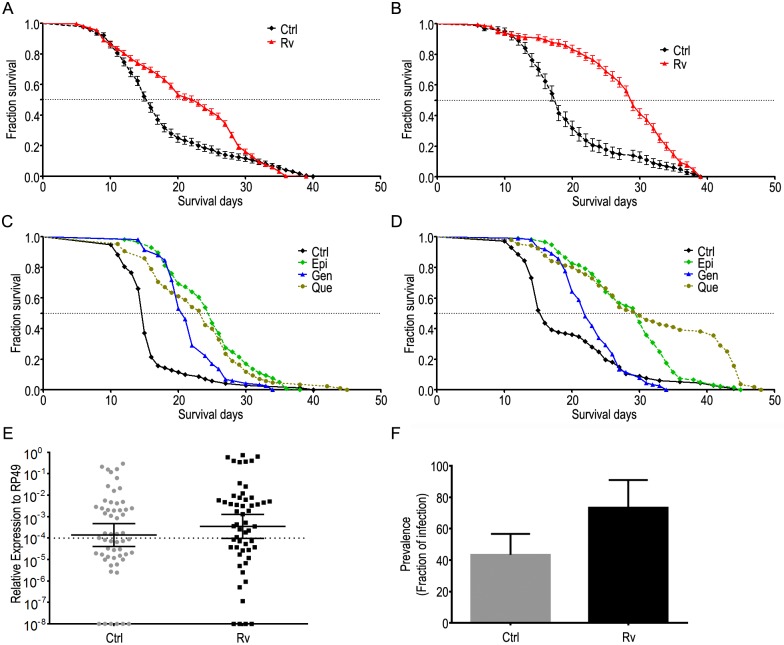
Polyphenols increase mosquito lifespan while still allowing dengue virus infection. (**A-D**) Effect of polyphenols on male (**A, C**) and female (**B, D**) lifespan. The 50% mean lifespan is indicated by the horizontal dotted line. (**E**) Dengue virus RNA from mosquitoes fed or not before infection with Rv. (**F**) Percentage of dengue-infected mosquitoes. Ctrl—Control, Epi—100 μM epi-gallo-catechin-gallate; Gen—100 μM genistein; Que—100 μM quercetin. (Error bars,s.e.m., n = 3 experiments with 200 mosquitoes in each cage, p<0.05 in each survivor curve compared with control as calculated by Mantel-Cox and Gehan-Breslow-Wilcoxon test).

Results were analyzed using a t-test (when only two conditions were compared) or a one-way ANOVA (when more than two conditions were compared), followed by Dunnett's (one control) or Bonferroni’s post-test (more than one control). In Fig 1, geometric means were considered different when a statistical significance level (α) lower than 0.05 was found using the Mann-Whitney test for comparison. All experiments were performed at least 3 times, and the analyses were performed using the GraphPad Prism 6 software.

### Phylogenetic analysis

AMPK catalytic subunit (α) sequences were obtained from three different databases: a) Kinbase (http://kinase.com/kinbase), Vector base (http://www.vectorbase.org/), and the NCBI protein database (http://www.ncbi.nlm.nih.gov/protein). All sequences in the CAMK/CAMKL/AMPK subfamily at Kinbase were included. Vector base and the NCBI protein database were blasted using AAEL007153 as the query to retrieve AMPKs from some organisms that were not included in Kinbase. All the results were manually inspected to confirm that sequences without previous annotation were AMPKs. Sequences were aligned using Muscle [32], and the phylogenetic trees were calculated with RAxML [33] using the JTT matrix and 1000 bootstrap replicates. The tree design used iTol [34].

### Imaging autophagy in mosquito midgut

We adapted this technique from other established models [35]. Midguts were dissected in PBS and incubated in 1 μM Lysotracker Red (Invitrogen) in PBS for 10 minutes. Midguts were washed 3 times with PBS and transferred to glass slides. Samples were visualized and photographed on a fluorescence microscope (Axioskop, Zeiss). The densitometry of the images was conducted using Image J. For transmission electron microscopy analysis, midguts were fixed with 2.5% glutaraldehyde in 0.1 M Na-cacodylate buffer (pH 7.2) at room temperature for 1 h at 25°C and post-fixed with a solution of 1% OsO_4_, 0.8% potassium ferricyanide and 2.5 mM CaCl_2_ in the same buffer for 1 h at 25°C. The samples were dehydrated in an ascending acetone series and embedded in PolyBed 812 resin. Ultrathin sections were stained with uranyl acetate and lead citrate and examined in a Jeol 1011 transmission electron microscope (Tokyo, Japan) at FIOCRUZ Electron Microscopy Platform.

## Results

### Polyphenols enhance mosquito lifespan and do not affect its refractoriness to dengue virus

To assess whether polyphenol dietary intake affects *A*. *aegypti* lifespan, we evaluated mortality rates of mosquitoes of both sexes fed *ad libitum* on a sugar solution containing different polyphenols at a concentration of 100 μM (Fig 1). Insects treated with polyphenols displayed a marked increase in average lifespan, for both males (Fig 1A and 1C and Table 1) and females when compared with controls (Fig 1B and 1D and Table 1). In males fed a sugar solution containing Rv, the average lifespan increased by 52.9%, from 15.1 days (± 2.60) to 23.2 days (± 2.94) and that of females increased by 49.6%, from 20.0 (± 0.50) to 29.9 (± 2.18) (Table 1). Polyphenol-fed mosquitoes fed blood containing dengue virus were still prone to infection (Fig 1E and 1F).

**Table 1:** Summary data of the effects of polyphenols on mosquito lifespan.

Mean Survival Days			Maximum Survival Days		
Condition	Male	Female	Condition	Male	Female
**Ctrl**	15.17 ± 2.60	20.00 ± 0.50	**Ctrl**	36.75 ± 2.66	37.25 ± 3.09
**Rv**	23.21 ± 2.94	29.92 ± 2.18	**Rv**	38.00 ± 0.58	38.00 ± 2.85
**Epi**	25.00 ± 1.00	28.00 ± 3.50	**Epi**	36.00 ± 1.00	38.50 ± 5.50
**Gen**	24.50 ± 1.50	22.00 ± 1.50	**Gen**	31.00 ± 2.00	31.50 ± 1.50
**Que**	24.25 ± 1.25	26.00 ± 3.00	**Que**	38.50 ± 5.50	40.00 ± 7.00
**AbMix**	20.25 ± 2.25	21.75 ± 3.25	**AbMix**	40.00 ± 4.00	39.00 ± 1.00

### Mosquito AMPK pathway is activated upon polyphenol ingestion

**Fig 2:**
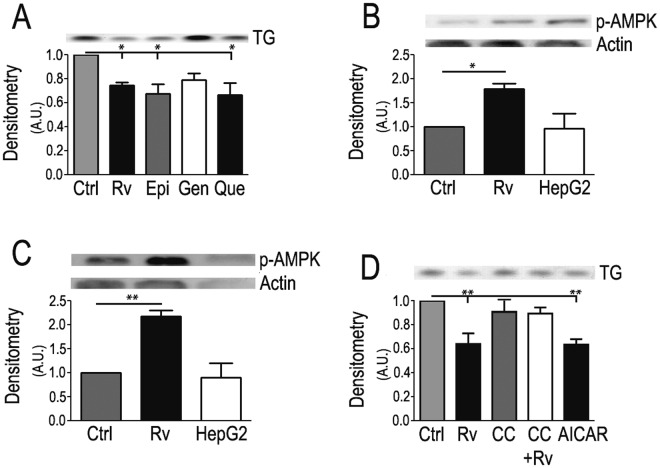
Dietary polyphenols decrease lipid accumulation in mosquitoes through AMPK activation. (**A**) Densitometric analysis of triacylglycerol content measured by TLC. (**B**) Densitometric analysis of western blots for midgut p-AMPK. (**C**) Densitometric analysis of western blots for fat body p-AMPK. (**D**) Densitometric analysis of triacylglycerol levels as measured by TLC in mosquitoes fed as indicated. Actin was used as loading control in panels B and C. (HepG2) HepG2 cells are a positive control for pAMPK antibody. Insets show representative images of either TLCs or blots. Ctrl—control; Rv—100 μM Rv; Epi—100 μM epi-gallo-catechin-gallate; Gen—100 μM genistein; Que—100 μM quercetin; CC—100 μM Compound C; CC+Rv—100 μM Compound C and 100 μM Rv; AICAR—1 mM AICAR. *—p<0.05; **—p<0.01, as calculated by one-way ANOVA with Tukey post test. (Error bars, s.e.m., n = 3 experiments).

Polyphenols are widely known as activators of the AMP-dependent protein kinase (AMPK) pathway [36]. AMPK is a master regulator of metabolism that usually leads to the downregulation of triglyceride (TG) synthesis. The *A*. *aegypti* AMPK catalytic subunit (α) shows a consistent and coherent position in the Animalia clade (S1 Fig). We thus quantified lipid content and AMPK activation in polyphenol-fed insects. Thin-layer chromatography of Rv-fed mosquitoes to determine TG content showed that all tested polyphenols similarly suppressed lipid content (Fig 2A). AMPK activation classically occurs by phosphorylation of Threonine 172 in the catalytic subunit [36]. This phosphorylation is recognized by specific antibodies in Western blots. Rv-fed insects displayed a higher level of AMPK phosphorylation in both the midgut (Fig 2B) and fat body (Fig 2C). It is important to highlight that Rv diet enhances this phosphorylation step as compared to controls. Thus mosquitoes continuously fed on polyphenol diets displayed a persistent, higher level of AMPK phosphorylation and activity. Accordingly, when insects were fed Compound C, an inhibitor of the AMPK pathway, the decrease in TG content promoted by Rv was blocked (Fig 2D). AICAR (5-aminoimidazole-4-carboxamide ribonucleotide), a classical activator of AMPK, decreased the total TG content to levels similar to those obtained in the Rv-fed mosquitoes (Fig 2D).

### Mosquito midgut bacteria population is controlled by polyphenol-induced autophagy

**Fig 3:**
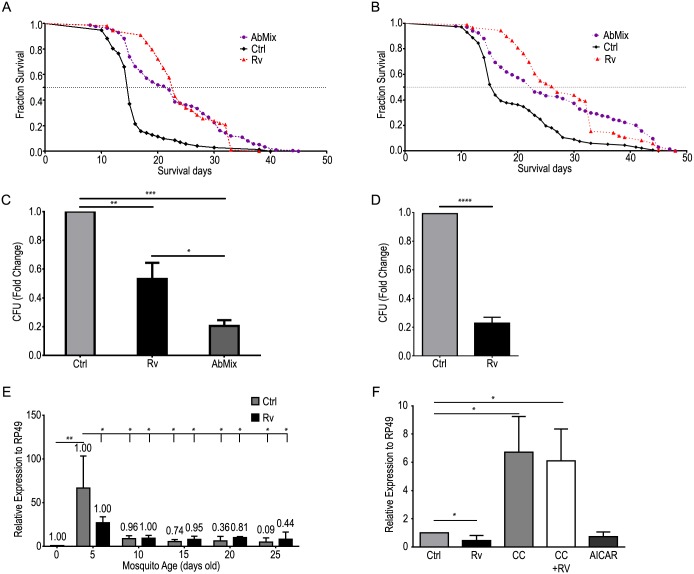
Rv-mediated activation of the AMPK pathway reduces mosquito midgut microbiota and increases lifespan. (**A, B**) Effect of polyphenols and antibiotics on male (**A**) and female (**B**) lifespan. Horizontal dotted lines indicate the mean lifespan. (**C, D**) Colony-forming units (CFU) from the midgut of mosquitoes obtained from two geographically isolated colonies (C, Rio de Janeiro, RJ, Brazil; D, Campos dos Goytacazes, RJ, Brazil). (**E**) 16S rRNA levels of mosquito midguts obtained from insects fed in the presence or absence of Rv. Numbers above each bar indicate the proportion of live mosquitoes observed in the experiments depicted in Fig 3B (Fraction survival). (**F**) 16S rRNA gene levels in the midguts of females reared under each dietary condition. Ctrl—control; Rv—100 μM Rv; CC—100 μM Compound C; CC+Rv—100 μM Compound C and 100 μM Rv; AICAR—1 mM AICAR; AbMix– 10 U/mL penicillin, 10 U/mL streptomycin and 15 U/mL gentamicin. *—p<0.05; **—p<0.01; ***—p<0.001; ****—p<0.0001, as calculated by one-way ANOVA with Tukey post test (3D as calculated by Student’s t-test). (Error bars, s.e.m., n = 3 experiments with 200 mosquitoes in each cage in panels a and b, p<0.05 in each survivor curve compared with control as calculated by Mantel-Cox and Gehan-Breslow-Wilcoxon test).

The larva-pupa-adult metamorphosis represents a challenge for the mosquito´s innate immune system with regard to the midgut microbiota [37]. The control of midgut microbiota relies on the activation of diverse immune pathways. The observed polyphenol-mediated increase in average lifespan may, in part, result from an adjustment of immune pathways that regulate bacterial loads in the midgut. This hypothesis is supported by the longer lifespan that results from adding an antibiotic mix to the diets (Fig 3A and 3B). We therefore evaluated, using different techniques, the total number of bacteria in the midgut of female mosquitoes obtained from geographically isolated colonies. Rv treatment resulted in reduced bacterial populations at day 5 compared with controls (Fig 3C and 3D). Bacterial loads were then analyzed throughout the mosquito lifespan (Fig 3E). In both the control and Rv- fed groups mosquitoes with a longer lifespan displayed reduced midgut bacterial loads. However, in polyphenol-fed populations there were more individuals with lower bacterial loads (Fig 3A, 3B and 3E). Thus, older mosquitoes are most likely selected in mosquito populations that had access to polyphenols early in their adult life (Fig 3A and 3E). Also, the effect of Rv on midgut microbiota was mimicked by the AMPK activator AICAR (Fig 3F). Interestingly, a 6-fold increase in bacterial proliferation was observed in mosquitoes treated with the AMPK inhibitor Compound C in either the presence or absence of Rv (Fig 3F). However, Rv treatment slightly decreased the bacterial populations when bacteria were isolated from the midgut and treated with Rv *in vitro* (S2 Fig). These results suggest that the decrease in the midgut bacterial populations in polyphenol-fed mosquitoes was caused by the activation of the mosquito immune responses. We then assayed midgut reactive oxygen species (ROS) production and the expression of anti-microbial peptides (AMPs) in the midgut and fat body [27]. Neither ROS production nor AMPs expression was significantly affected by Rv treatment (S3 Fig).

### Polyphenol diet downregulates the expression apoptotic genes and activates autophagy in mosquito midgut

**Fig 4:**
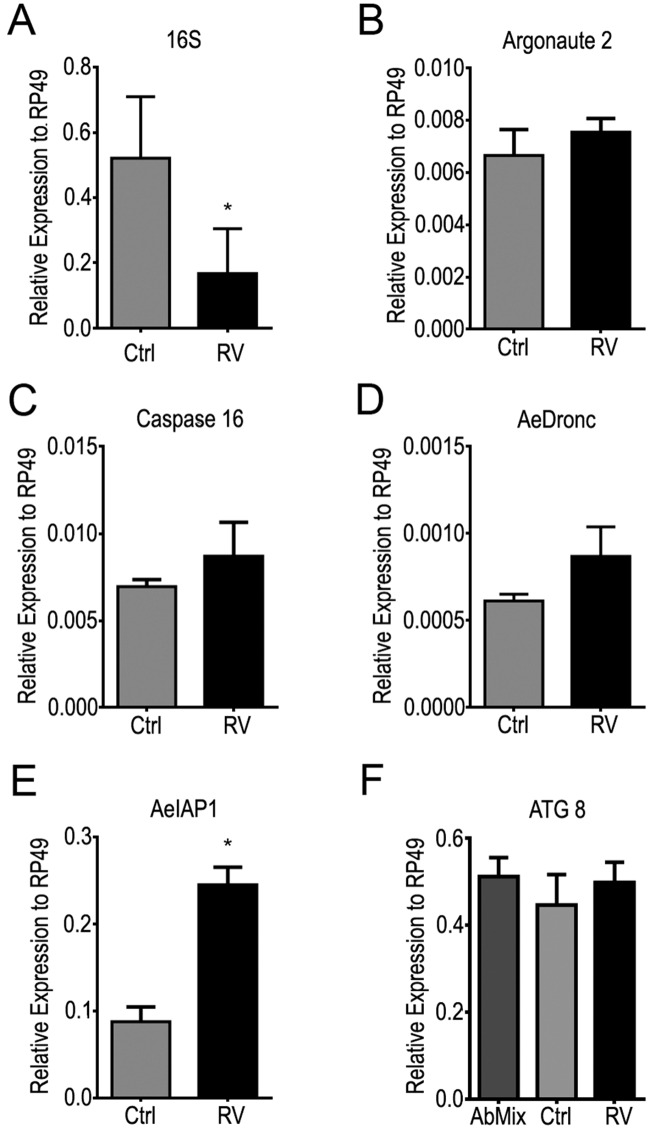
Rv treatment downregulates apoptotic pathway. Adult mosquitoes were reared until six days old under each dietary condition. The midguts of female mosquitoes were dissected and homogenized in TRIzol, and total RNA was extracted. These samples were used to perform qPCR for the genes: (**A**) 16S, (**B**) ARGONAUTE 2, (**C**) CASPASE 16, (**D**) AeDRONC, (**E**) AeIAP1 and (**F**) ATG8. Data show means and standard error of at least three independent experiments. Ctrl—0.05% ethanol plus 10% sucrose; Rv—100 μM Rv in 0.05% ethanol plus 10% sucrose; AbMix– 10% sucrose, 10 U/mL penicillin, 10 U/mL streptomycin and 15 U/mL gentamicin. *—p<0.05; **—p<0.01; ***—p<0.001; ****—p<0.0001, as calculated by Student’s t-test (F as calculated by one-way ANOVA with Tukey post test). (Error bars, s.e.m., n = 3 experiments).

We next examined the involvement of apoptosis in the control of mosquito midgut microbiota through the expression of AeDRONC, inhibitor of apoptosis (AeIAP1), Caspase 16 and ARGONAUTE 2 (Fig 4). In Rv-fed mosquitoes the levels of 16 S were downregulated compared to the control (Fig 4). Also, the expression of AeIAP, but not of AeDRONC, Caspase 16, Argonaute was stimulated by Rv treatment (Fig 4D and 4E). Such results indicate the polyphenol-fed mosquitoes display a downregulation of apoptotic pathway. However, in the same conditions the levels of mRNAs for ATG8 were not affected even in antibiotic treated insects.

**Fig 5:**
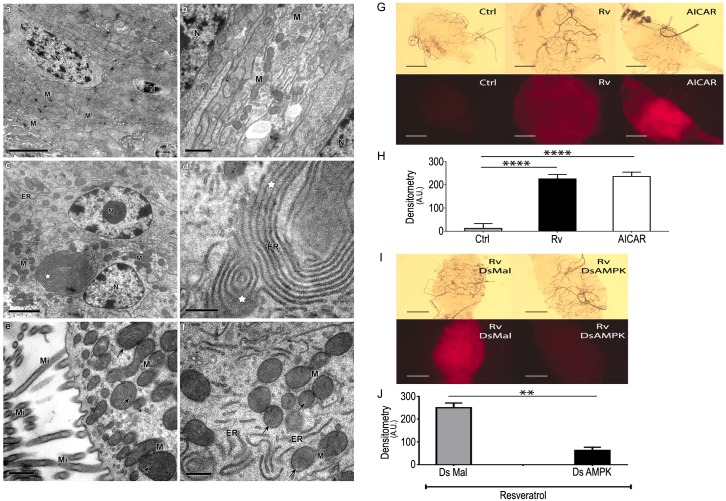
Rv triggers autophagy in mosquito midgut. **(A, B**) Typical ultrastructural appearance of mosquito midgut fed on a control diet. (**C-F**) Midguts from insects fed on Rv. White stars indicate the formation of concentric membrane structures. Black arrows indicate preserved mitochondrial morphology and normal cristae aspects. N: nucleus; M: mitochondria; Mi: microvilli. Bars in panels A and C: 2 μm. Bars in panels B, D-F: 0.5 μm. (**G**) Midgut images obtained under a fluorescence microscope after incubation with LysoTracker Red (upper panels, DIC) (lower panels, fluorescence). (**H**) Densitometry of LysoTracker fluorescence images shown in panel G. (**I)** Images obtained as in panel G following the silencing of mosquito AMPK (DsAMPK) or an unrelated protein (DsMal). Insects were kept on the indicated Control (Ctrl), Rv or AICAR diets. **(J**) Densitometry of LysoTracker fluorescence images shown in the lower part of panels I. Rv—100 μM Rv; AICAR—1 mM AICAR. **—p<0.01; ****—p<0.0001 as calculated by one-way ANOVA with Tukey post test (I) and by Student’s t-test (J). Data in panels I, J represent means and s. e. m. (n = 3).

AMPK regulates immunity through the activation of autophagy [24,38–40]. Autophagy is a highly organized process that allows for the degradation of damaged organelles or protein aggregates in vesicles known as autophagosomes. Furthermore, AMPK-mediated autophagy is a pathway that controls bacterial proliferation in several models [41]. Therefore, we investigated whether AMPK activation by polyphenols would ultimately lead to an autophagy-mediated decrease in mosquito midgut bacteria. Through electron microscopy we compared the midgut epithelia of control mosquitoes and polyphenol-fed insects (Fig 5). In insects fed polyphenols, several autophagy-related concentric membrane structures that were not seen in controls were detected throughout the cytosol of the midgut epithelia (Fig 5C–5F). Polyphenol treatment also resulted in the appearance of endoplasmic reticulum profiles surrounding cellular structures, especially mitochondria (Fig 5D and 5F). Additionally, typical mitochondrial morphology, including aspects of normal cristae, was observed (Fig 5E and 5F). We next evaluated the role of AMPK in autophagosome formation (Fig 5G–5J). Rv- and AICAR-treated midguts displayed large numbers of active autophagosomes (Fig 5G and 5I). AMPK RNAi silencing partially suppresses autophagy in mosquito midgut (S4 Fig) in sucrose-fed mosquitoes. However, in Rv-treated mosquitoes silencing of AMPK completely abolishes the autophagy promoted by polyphenol ingestion (Fig 5H and 5J).

## Discussion

The present study demonstrates that the polyphenol rich diet triggers an autophagy pathway that leads to a reduction in midgut microbiota. We also show that this polyphenol rich diet leads to an enhancement of vector lifespan [42] (Figs 1–5). Polyphenols, however do not alter the susceptibility to dengue virus infection. Thus, molecular mechanisms that enhance average lifespan do not impair pathogen infection and transmission. Also, it is likely that lifespan-promoter effects mediated by polyphenols are confined to some Diptera species since they were also reported in *Drosophila* [43] but not in *Anopheles* [44]. Also, lifespan promotion in *Drosophila* relies on the activation of AMPK-autophagy axis [45,46]. Such aspects of Diptera signalling must be investigated at the genomic level in the future and may be due to differences in signalling pathways accumulated during the evolution of this group of insects [47]. In nature, mosquitoes that continuously feed on polyphenol-containing plant fluids may exhibit a more efficient establishment of autophagic pathways leading to a decrease in gut bacteria in the early days of their adult life. The decrease in bacterial population upon polyphenol treatment occurs at different levels in different mosquito colonies (Fig 3C and 3D) but does not impose logarithmic losses in bacterial population. Such changes proportionally impose significant increases in average mosquito lifespan (Figs 1–5). Similar modulation of midgut bacterial levels and the consequences for vector lifespan were also reported in mosquitoes fed with blood enriched with antibiotics [24]. In the present study midgut bacterial populations were observed only after pharmacological inhibition of AMPK (Figs 3F and 5). Also, analysis of bacterial population throughout mosquito lifespan showed that long-lived insects display lower levels of microbiota (Fig 3E). Altogether, these results indicate that bacterial loads are a major determinant of mosquito lifespan and that polyphenol effects rely on the sustained activation of AMPK pathway. The present results are based on our view that bacteria growing on this medium will behave the same as the unrecorded bacteria that have not grown, or have been out-competed on this media or conditions used. Thus, the final extent of vector lifespan is probably due to an enhancement of the interplay between metabolism and immunity promoted by dietary components. It is likely that in the early days of terrestrial life the composition of microbiota instead of its dimension may be a more precise indicative of mosquito lifespan. Future analysis of mosquito microbiota after the ingestion of polyphenol-diets may shed light on the relevant bacterial species that are associated with an increase of average lifespan and that ultimately determine an increase in total lifespan. Such combined effects lead to a population with a greater number of mosquitoes that display an increased lifespan. For these populations, more male mosquitoes are available for reproduction and females can wait a longer period before seeking their first blood meal. As a result, the final number of mosquitoes feeding on blood and transmitting pathogens such as dengue is likely greater whenever polyphenols are present in their diets.

Polyphenol effects are usually associated with their ability to mimic calorie restriction [38]. In fact a detailed comparison of the metabolic effects of calorie restriction and Rv supplementation demonstrated a parallel between these two interventions [38]. Most effects of calorie restriction reported in the literature are associated with the optimization of energy expenditure and metabolism, mitochondrial efficiency, a decrease in oxidative damage and improvement of whole-body insulin sensitivity [38]. Rv-fed *Drosophila* also display an extension of average lifespan similar to that found with calorie restriction [43]. Also, the lifespan promotion in *Drosophila* induced by β-guanidinopropionic acid elevated the expression of phospho-T172-AMPK levels while RNAi silencing of this enzyme attenuated the expression of autophagy-related proteins and lifespan extension [46]. Altogether these data indicate that the lack of an additive effect of Rv and calorie restriction suggests that this polyphenol extends lifespan through a mechanism related to calorie restriction, probably relying on the similar steps of activation of AMPK pathway [43].

Several different high-throughput molecular approaches have been used in attempts to detail the recurrent downstream signalling elements triggered by lifespan promoters and their connection with the autophagy machinery [40,48]. It is commonly thought that polyphenol enhancement of lifespan through the autophagic pathway requires the nicotinamide adenine dinucleotide-dependent deacetylase sirtuin 1 (SIRT 1). Nevertheless, similar effects are triggered by spermidine through a SIRT 1-independent pathway [48]. Despite this major difference in the mechanism of autophagy initiation, Rv and spermidine signalling pathways converge to a common mechanism that relies on profound modifications in the acetylproteome [48]. Spermidine and Rv affected the phosphorylation status of multiple kinases and also induced the differential acetylation of 375 proteins, 170 of which are part of the human autophagy protein network. Further phosphoproteomic analysis of cells treated with the autophagy inducers spermidine and Rv revealed the presence of over 800 different phosphorylation sites that were co-regulated in response to the treatment with such drugs [40]. Curiously, dephosphorylation events were more frequent in spermidine- and Rv-treated cells and several of them occurred as a result of protein kinase inactivation. Also, Rv and spermidine treatment induced a wide array of kinase-dependent signals with more than 109 dephosphorylation events occurring within kinases or direct kinase regulators involved in the autophagy response [40]. These findings strongly implicate signalling pathways involved in autophagic response as key modulators of lifespan.

Calorie restriction is a non-pharmacological promoter of lifespan extension. Increased expression SIRT2 or suppression of p53 promotes lifespan extension in *Drosophila* in a calorie restriction-dependent fashion [49,50]. A multiple comparison approach based on whole-genome transcriptional arrays was used to identify genes and pathways involved in calorie restriction lifespan extension in *Drosophila*. A three-way comparison of flies subjected to calorie restriction with SIRT 2 and p53 long-lived flies demonstrated that 20 genes are upregulated in both groups, including *takeout*, a gene that has juvenile hormone (JH) binding-like domains and is capable of extending fly lifespan [51]. Also, the comparison between calorie- restriction flies and Rv-fed insects showed a huge overlap (81%) of common genes undergoing variations in expression following the treatments [52,53]. Thus *takeout* seems to be a central gene involved in all lifespan promotion experiments. Taken together with the suppression in the expression of genes usually downregulated by JH treatment, this finding suggests that JH signalling is downregulated in long-lived insects. Knockout of the corpora allata in *Drosophila* also reduces JH production and is a sufficient condition to extend lifespan [54].

The effect of diet restriction during either larval or adult stages was previously demonstrated in *A*. *aegypti*. Females fed either a single blood meal or none survived 30–40% longer [55]. These findings suggest a novel strategy to address the intracellular signalling routes responsible for lifespan extension in blood-fed mosquitoes. Diet restriction in mosquitoes decreased JH synthesis through the decrease in insulin signalling in *corpora allata*, specifically through the suppression of insulin-related genes such as insulin receptor, the Forkhead-box-binding protein and the translation initiation inhibitor eIF4E-binding protein [56]. Curiously, introduction of exogenous insulin into the mosquito diet reduced *Anopheles* sp. lifespan [57], while overexpression of Akt in the mosquito fat body enhanced vector lifespan [58]. These results indicate that polyphenol extension of average lifespan may rely on the downregulation of insulin pathway which classically relies on AMPK activation. In addition, the role of autophagy in the calorie restriction-induced lifespan and its interplay with insulin signalling remains to be demonstrated in mosquitoes. In *Drosophila* AMPK-mediated tissue specific mechanisms and autophagy induction operate to promote slow aging [45]. Thus insulin-mediated regulation of autophagy in mosquitoes may represent a unique scenario of AMPK regulation in blood-sucking insects and should be further investigated.

A phylogenomic study has suggested that insect colonization of a terrestrial habitat occurred roughly at the same time as plants [59]. Thus, during insect evolution, the first herbivorous and phytophagous species may have benefited from a polyphenol-induced increase in lifespan. This major benefit likely affected the ecological relationships among insect species and helped shape plant-insect interactions. Additionally, the lives of larvae that develop in water containing decomposing plants are shortened by such compounds [15]. Usually, polyphenols are toxic to mosquito larvae and their effect on the population structure has been addressed [60]. There is a lack of correlation between the genetic and environmental parameters, and local adaptation is a labile trait that occurs with great heterogeneity among different mosquito species [16]. Surprisingly, adult mosquitoes tolerate polyphenol ingestion and live for a considerable period continuously feeding on polyphenol-rich diets (Fig 1). A recent study reports that the presence of sugar sources in the environment modulates the dynamics of mosquito populations and their vector potential [61]. The preference of *Aedes* sp. for some ornamental plants species has been described [62], and garden plants have been tested for attractiveness to *Culex* sp. and *Aedes sp*. [63]. Altogether, our findings indicate that vegetation within and around urban areas may promote polyphenol-potentiated vector transmission. Thus the idea of eliminating or reducing polyphenol production in plants in urban areas should be investigated as a novel strategy for environmental reduction of dengue transmission.

## Supporting Information

S1 Table List of primers sequences. (TIF)

S1 Fig Phylogenetic tree of AMPK. AMPKα sequences were obtained from Kinbase, NCBI and VectorBase. Multiple alignments and tree construction were performed using Muscle and RAxML. Numbers inside the tree represent bootstrap support in 1000 replicates. Database identifiers are within parentheses. Black circles indicate sequences previously annotated as AMPK.
(TIFF)

S2 Fig Rv treatment in vitro does not kill bacterial colonies isolated from mosquito midgut. Mosquito gut bacteria were cultured in BHI and plated on Petri dishes (tissue from one mosquito per dish) together with paper discs as indicated. Discs were pre-soaked in one of the following solutions: #1–200 μg/mL rifampicin; #2–5% ethanol; #3 and #5–500 μM Rv; #4 and #6–50 μM Rv. The growth of bacterial colonies indicated at the bottom of each panel was evaluated and photographed after 48 h. Dark area around each disc paper indicates inhibition of the growth of bacterial colonies.
(TIF)

S3 Fig Polyphenols neither kill bacteria directly nor affect ROS and anti-microbial peptides production in mosquito midgut and fat body. The major bacterial strains isolated from female midguts were grown in liquid Muller-Hinton medium (A) or in liquid BHI medium (B), and their optical density was measured. Data show means and standard errors of at least three independent experiments. (C, D) Adult mosquitoes were reared until six days old under each dietary condition. The midguts and fat bodies of female mosquitoes were dissected and incubated in 5 μM dihydroethidium for 20 min, then images were obtained under a fluorescence microscope (C) and quantified by densitometry (D). Midguts (e) and fat bodies (f) from Rv- or antibiotic-treated mosquitoes were homogenized in TRIzol, and total RNA was extracted. These samples were used to perform qPCR for the genes Attacin, Cecropin, Defensin and Gambicin. Ctrl- control; Rv- resveratrol; Abmix- antibiotic mix.
(TIF)

S4 Fig RNAi silencing of AMPK partially suppresses midgut autophagy. Mosquito females 2–3 days after emergence were anesthetized by cold and injected with 300 ng of RNAi dsMAL or dsAMPK and held for 5–6 days on a, 10% sucrose diet. Midguts were dissected and incubated maintained in a solution 1 μM Lysotracker Red solution form in the same saline for 10 minutes. Tissues were washed 3 times with saline and then, observed and photographed in a fluorescence microscope (Axioskop, Zeiss). The densitometry of these images were analyzed on the program Image J. n = 4 experiments. ***—P < 0.0001, calculated using the t test student with Welch correction. (A) Midgut image obtained under a fluorescence microscope after incubation of a dsMAL-injected midgut with Lyso Tracker Red (inset panel, DIC); (B) Midgut image obtained under a fluorescence microscope after incubation of a dsAMPK-injected midgut with Lyso Tracker Red (inset panel, DIC); (C) Densitometry of LysoTracker fluorescence images obtained in the experiments shown on panels A and B, *—P < 0.0001, calculated using the t test student with Welch correction; (D) Quantification of RNAs on dsMAL and dsAMPK injected mosquitoes.
(TIF)
