# Supplementary figures and images for: Polyphenol-Rich Diets Exacerbate AMPK-Mediated Autophagy, Decreasing Proliferation of Mosquito Midgut Microbiota, and Extending Vector Lifespan
Source: PLoS Negl Trop Dis. 2016 Oct 12;10(10):e0005034. doi: 10.1371/journal.pntd.0005034 (PMC5061323; doi:10.1371/journal.pntd.0005034)

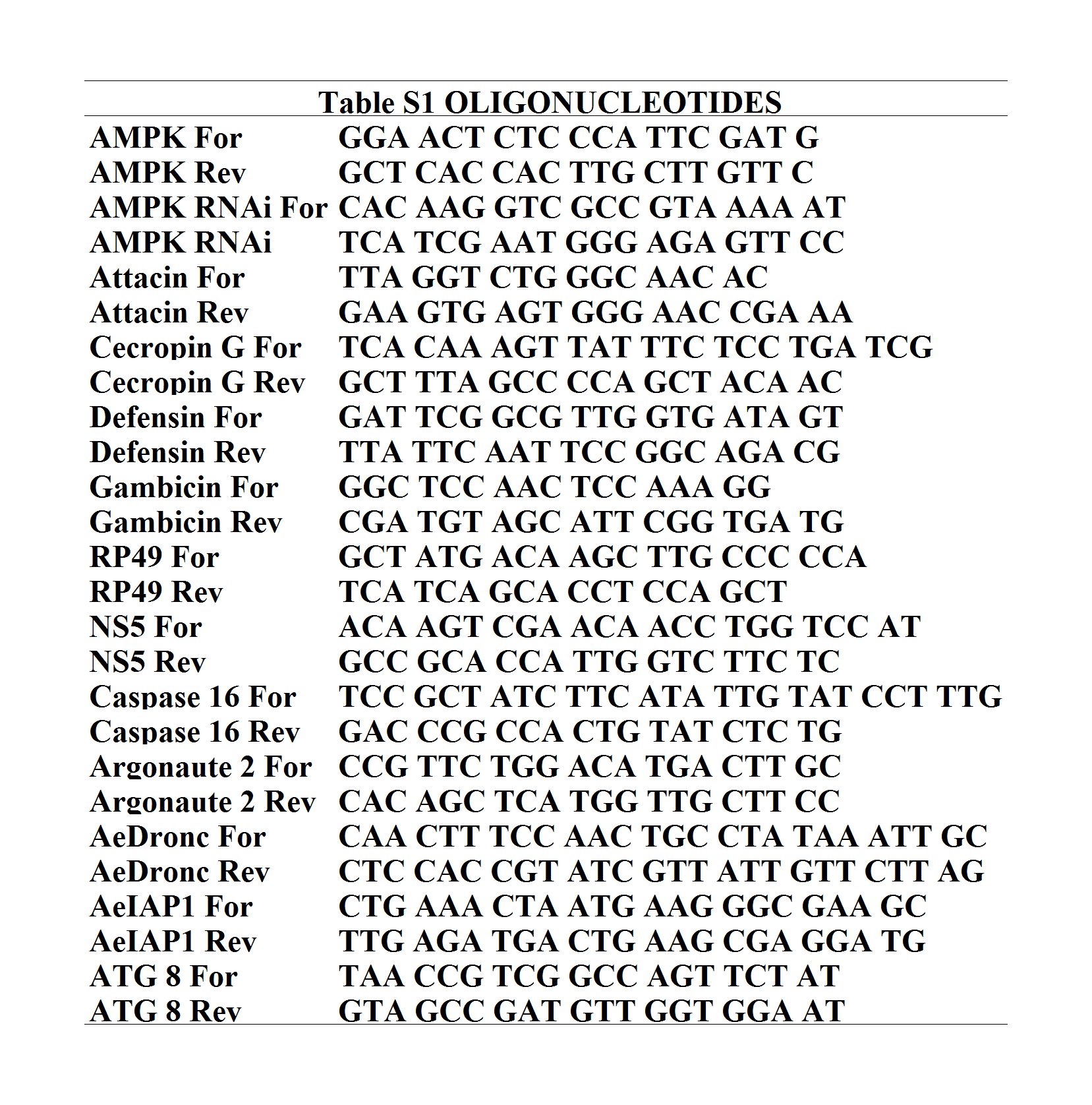

Supplement: S1 Table — (TIF) [file pntd.0005034.s001.tif]

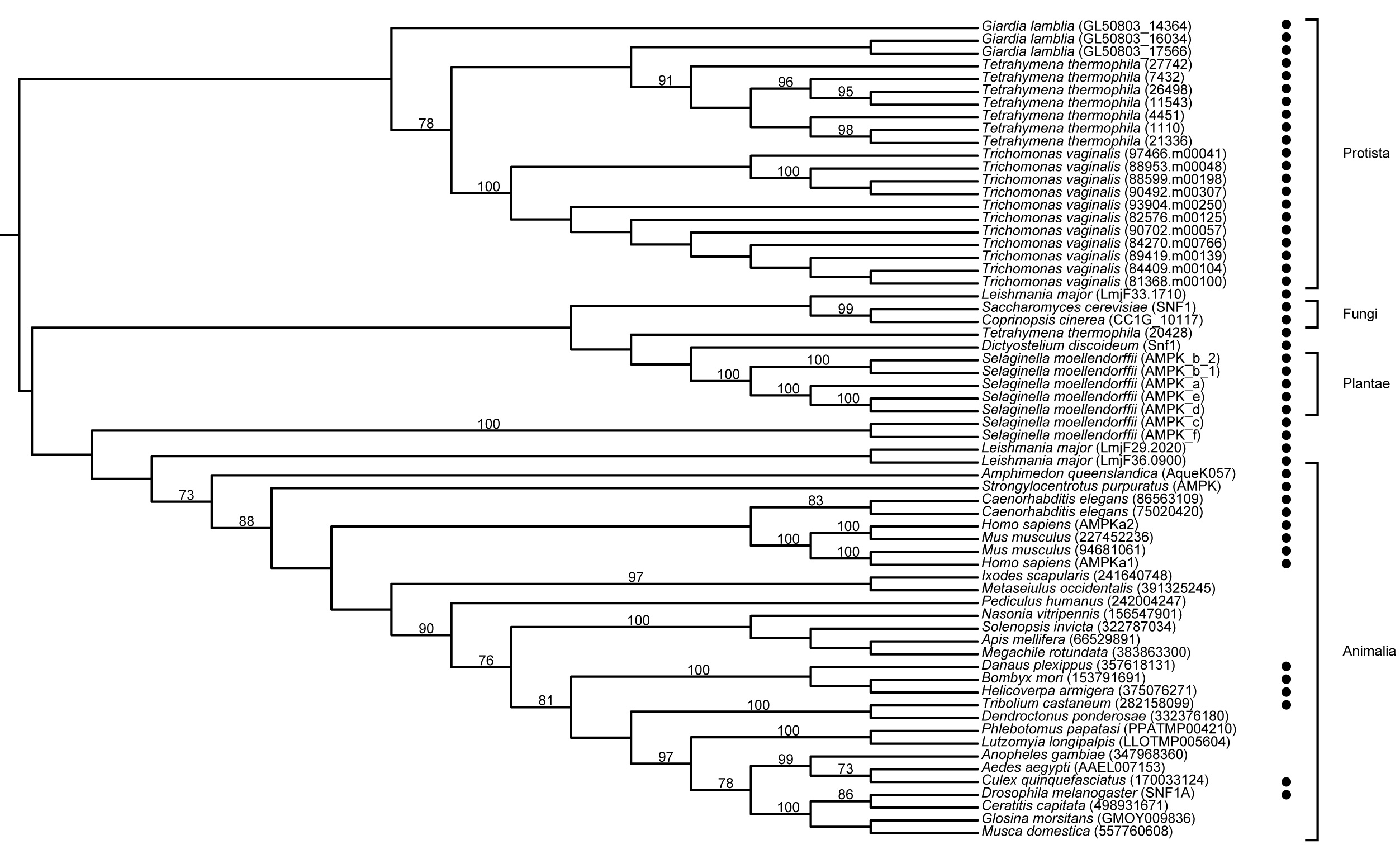

Supplement: S1 Fig — AMPKα sequences were obtained from Kinbase, NCBI and VectorBase. Multiple alignments and tree construction were performed using Muscle and RAxML. Numbers inside the tree represent bootstrap support in 1000 replicates. Database identifiers are within parentheses. Black circles indicate sequences previously annotated as AMPK. (TIFF) [file pntd.0005034.s002.tiff]

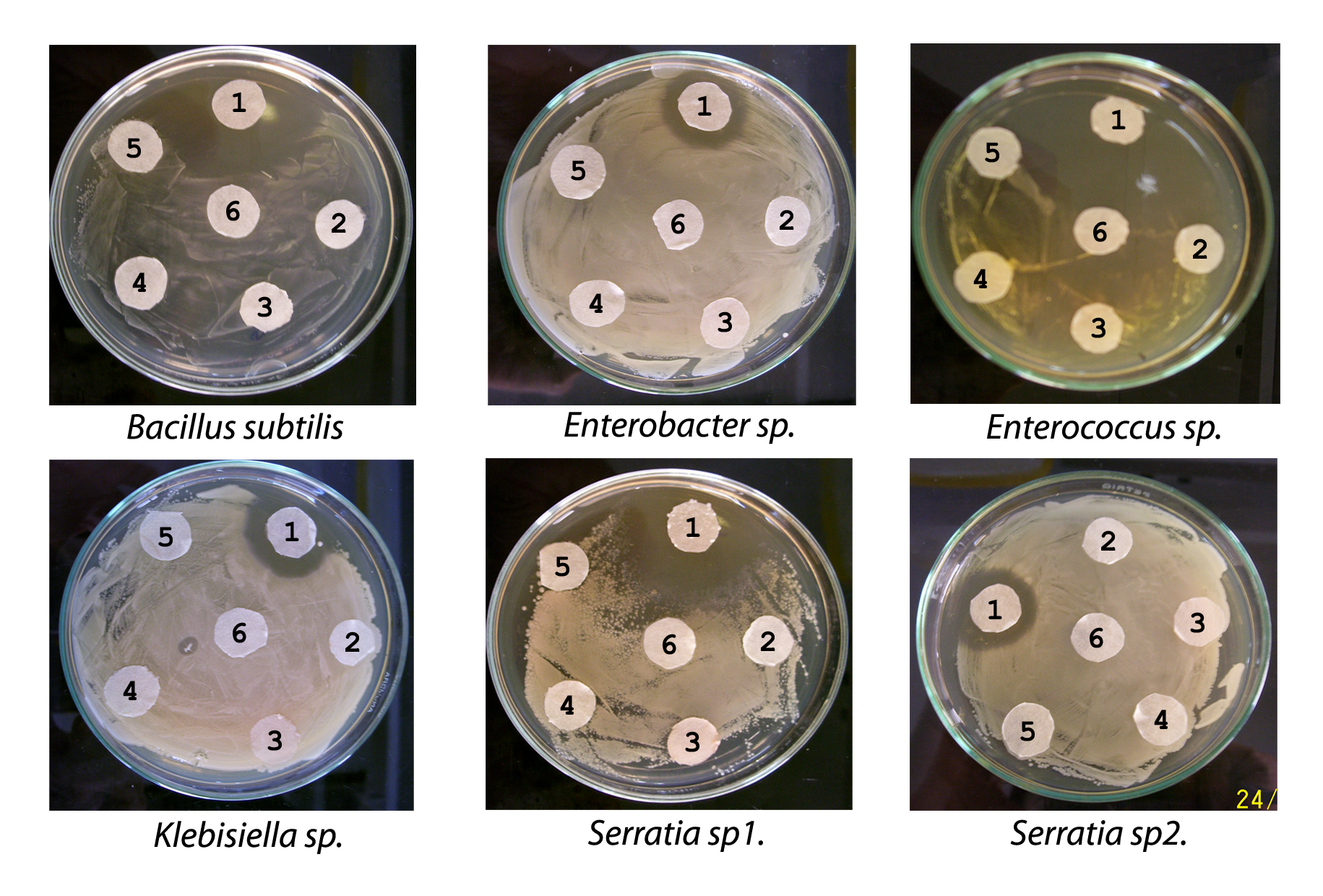

Supplement: S2 Fig — Mosquito gut bacteria were cultured in BHI and plated on Petri dishes (tissue from one mosquito per dish) together with paper discs as indicated. Discs were pre-soaked in one of the following solutions: #1–200 μg/mL rifampicin; #2–5% ethanol; #3 and #5–500 μM Rv; #4 and #6–50 μM Rv. The growth of bacterial colonies indicated at the bottom of each panel was evaluated and photographed after 48 h. Dark area around each disc paper indicates inhibition of the growth of bacterial colonies. (TIF) [file pntd.0005034.s003.tif]

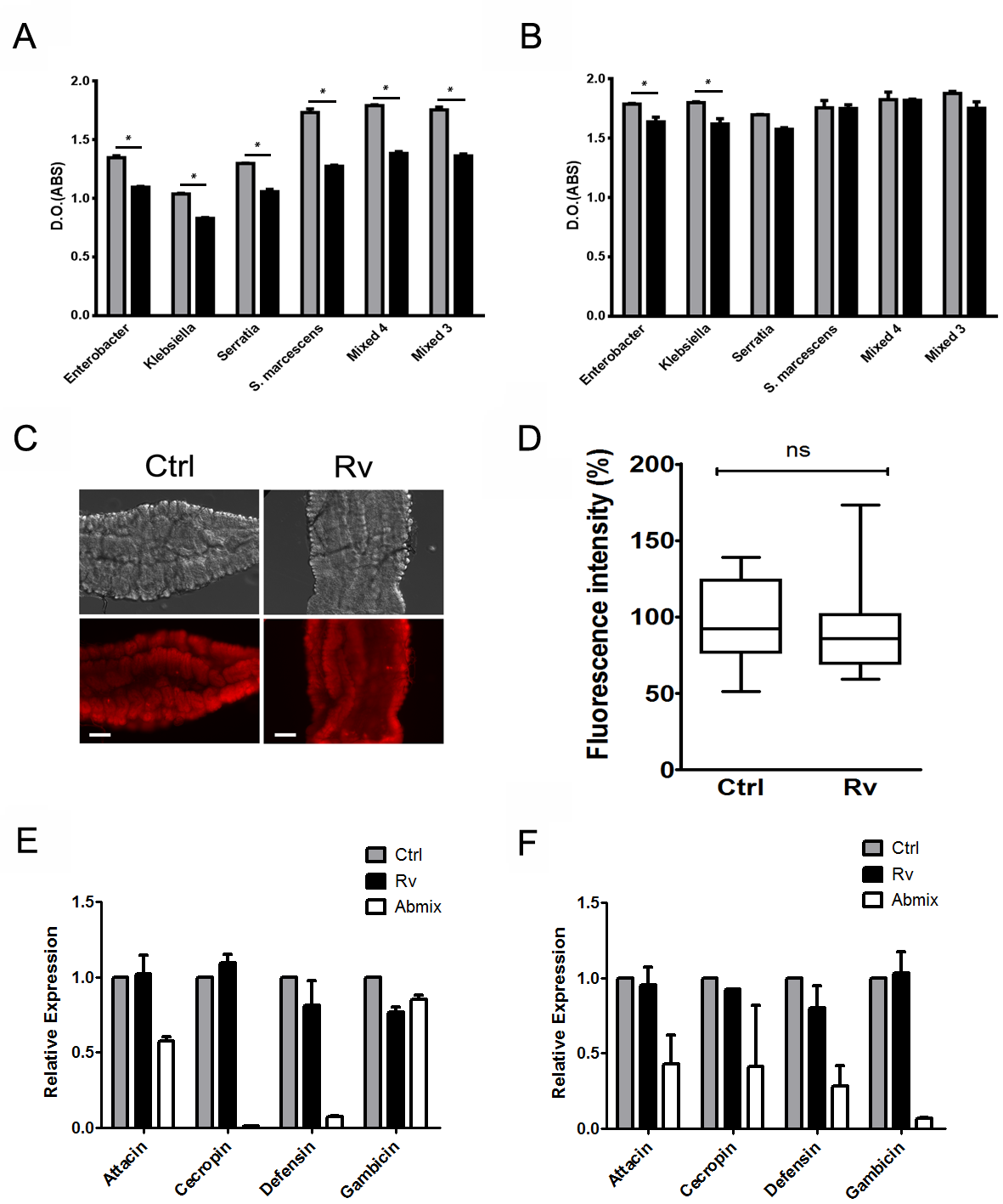

Supplement: S3 Fig — The major bacterial strains isolated from female midguts were grown in liquid Muller-Hinton medium (A) or in liquid BHI medium (B), and their optical density was measured. Data show means and standard errors of at least three independent experiments. (C, D) Adult mosquitoes were reared until six days old under each dietary condition. The midguts and fat bodies of female mosquitoes were dissected and incubated in 5 μM dihydroethidium for 20 min, then images were obtained under a fluorescence microscope (C) and quantified by densitometry (D). Midguts (e) and fat bodies (f) from Rv- or antibiotic-treated mosquitoes were homogenized in TRIzol, and total RNA was extracted. These samples were used to perform qPCR for the genes Attacin, Cecropin, Defensin and Gambicin. Ctrl- control; Rv- resveratrol; Abmix- antibiotic mix. (TIF) [file pntd.0005034.s004.tif]

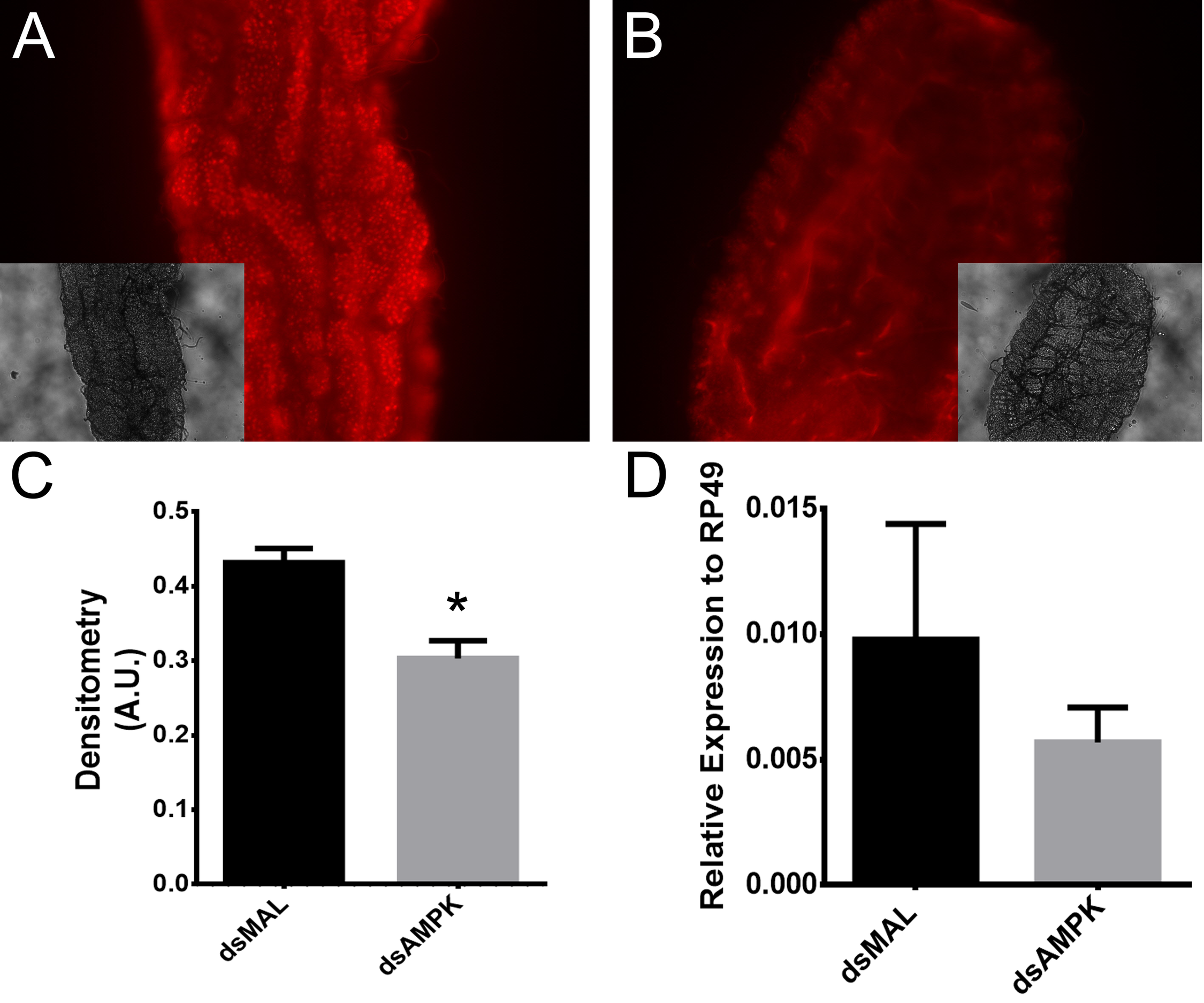

Supplement: S4 Fig — Mosquito females 2–3 days after emergence were anesthetized by cold and injected with 300 ng of RNAi dsMAL or dsAMPK and held for 5–6 days on a, 10% sucrose diet. Midguts were dissected and incubated maintained in a solution 1 μM Lysotracker Red solution form in the same saline for 10 minutes. Tissues were washed 3 times with saline and then, observed and photographed in a fluorescence microscope (Axioskop, Zeiss). The densitometry of these images were analyzed on the program Image J. n = 4 experiments. ***—P < 0.0001, calculated using the t test student with Welch correction. (A) Midgut image obtained under a fluorescence microscope after incubation of a dsMAL-injected midgut with Lyso Tracker Red (inset panel, DIC); (B) Midgut image obtained under a fluorescence microscope after incubation of a dsAMPK-injected midgut with Lyso Tracker Red (inset panel, DIC); (C) Densitometry of LysoTracker fluorescence images obtained in the experiments shown on panels A and B, *—P < 0.0001, calculated using the t test student with Welch correction; (D) Quantification of RNAs on dsMAL and dsAMPK injected mosquitoes. (TIF) [file pntd.0005034.s005.tif]
